# A High-Sensitivity Benchtop X-Ray Fluorescence Emission Tomography (XFET) System With a Full-Ring of X-Ray Imaging-Spectrometers and a Compound-Eye Collimation Aperture

**DOI:** 10.1109/TMI.2023.3348791

**Published:** 2024-05

**Authors:** Shubham Mandot, Elena M. Zannoni, Ling Cai, Xingchen Nie, Patrick J. La Rivière, Matthew D. Wilson, Ling Jian Meng

**Affiliations:** Department of Nuclear, Plasma and Radiological Engineering, University of Illinois at Urbana–Champaign, Urbana, IL 61801 USA; Department of Nuclear, Plasma and Radiological Engineering, University of Illinois at Urbana–Champaign, Urbana, IL 61801 USA; Department of Nuclear, Plasma and Radiological Engineering, University of Illinois at Urbana–Champaign, Urbana, IL 61801 USA; Department of Nuclear, Plasma and Radiological Engineering, University of Illinois at Urbana–Champaign, Urbana, IL 61801 USA; Department of Radiology, The University of Chicago, Chicago, IL 60637 USA; STFC-UKRI, Rutherford Appleton Laboratory, Didcot, OX11 0QX Oxfordshire, U.K.; Department of Nuclear, Plasma and Radiological Engineering and the Beckman Institute for Advance Science and Technology, University of Illinois at Urbana–Champaign, Urbana, IL 61801 USA

**Keywords:** X-ray fluorescence emission tomography, molecular imaging, XFCT, emission tomography

## Abstract

The advent of metal-based drugs and metal nanoparticles as therapeutic agents in anti-tumor treatment has motivated the advancement of X-ray fluorescence computed tomography (XFCT) techniques. An XFCT imaging modality can detect, quantify, and image the biodistribution of metal elements using the X-ray fluorescence signal emitted upon X-ray irradiation. However, the majority of XFCT imaging systems and instrumentation developed so far rely on a single or a small number of detectors. This work introduces the first full-ring benchtop X-ray fluorescence emission tomography (XFET) system equipped with 24 solid-state detectors arranged in a hexagonal geometry and a 96-pinhole compound-eye collimator. We experimentally demonstrate the system’s sensitivity and its capability of multi-element detection and quantification by performing imaging studies on an animal-sized phantom. In our preliminary studies, the phantom was irradiated with a pencil beam of X-rays produced using a low-powered polychromatic X-ray source (90kVp and 60W max power). This investigation shows a significant enhancement in the detection limit of gadolinium to as low as 0.1 mg/mL concentration. The results also illustrate the unique capabilities of the XFET system to simultaneously determine the spatial distribution and accurately quantify the concentrations of multiple metal elements.

## I. INTRODUCTION

In recent years, there has been a growing interest in the therapeutic applications of metal-based drugs and metal nanoparticles due to their favorable pharmacokinetic and pharmacodynamic properties in anti-tumor treatment [1], [2], [3], [4]. An example is the use of gadolinium nanoparticles (GdNP) as radiation therapy agents owing to their excellent radio-sensitizing efficiency and strong Auger electron emission properties [5], [6], [7], [8], [9]. Matsumoto et al. [10] have demonstrated the efficacy of gadolinium-loaded mesoporous silica nanoparticles (Gd-MSN) in destroying tumor spheroids prepared from ovarian cancer cells expressing green fluorescent proteins (GFP). In the study, they also tested the cytotoxicity of the Gd-MSN and found no toxic effect on human embryonic or ovarian cells up to 0.2 mg/mL. Gold nanoparticles (AuNP) are another example of a therapeutic agent loaded with a high atomic number (high-Z) element that offers promising cancer imaging and therapeutic potential [11], [12], [13], [14], [15], [16]. However, most of the studies reported so far with high-Z elements as therapeutic agents rely on *ex vivo* analysis and/or optical imaging techniques for quantification and biodistribution imaging of the metal elements [7], [8], [9], [10], [11]. These optical techniques, while offering high sensitivity and spatial resolution, are limited by shallow imaging depths due to high optical absorption and scattering.

X-ray fluorescence computed tomography (XFCT), on the other hand, uses X-ray fluorescence (XRF) photons emitted from metals upon X-ray irradiation to image and quantify the biodistribution of high-Z elements [17], [18], [19], [20], [21], [22], [23], [24], [25], [26], [27], [28], [29], [30], [31], [32], [33], [34], [35], [36], [37], [38], [39], [40], [41], [42], [43], [44]. This imaging modality helps to overcome the limitations posed by optical imaging techniques owing to the high penetrability of X-rays with a trade-off in sensitivity. We have previously proposed to use a SPECT-inspired system configuration for XFCT imaging [17], [18], [19], [45], in which the emitted fluorescence photons are detected using position-sensitive detectors coupled to collimating apertures. In order to further improve the sensitivity of the XFET approach, we have also incorporated the artificial compound-eye camera design as we have previously demonstrated for ultrahigh-performance SPECT imaging applications [46], [47].

Over the last several years, significant efforts have been made to develop benchtop XFCT systems capable of imaging molecular probes containing high-Z elements with high sensitivity. Some of the early developments of XFCT setups and algorithms utilized expensive synchrotron technology [20], [21], [22], [23], [24], [25], [26], which provides a high signal-to-noise ratio (SNR) but hinders the application of XFCT in routine biomedical imaging. In the last decade, many studies using both K-shell and L-shell X-ray fluorescence have shown promising detection limits of less than 5 mg/mL for various elements, including gold, gadolinium, and platinum nanoparticles with commercially available clinical X-ray sources [27], [28], [29], [30], [31], [32], [33], [34], [35]. However, in most benchtop XFCT systems developed so far, a single thermoelectrically cooled cadmium telluride (CdTe) detector placed perpendicular to the X-ray beam has been used to perform imaging studies. XFCT is inherently constrained due to the low SNR, high X-ray dose requirement, and long acquisition times, resulting from the intrinsically small photoelectric cross-section of metals and high background level from Compton scattering of the X-rays. Several novel collimator geometries, including multi-pinhole, parallel, converging, multi-slit, and more, have been proposed along with pencil, sheet, and cone-beam X-rays to speed-up the acquisition time and improve image quality and sensitivity [17], [18], [19], [36], [45]. Dunning and Bazalova-Carter performed a simulation study with different collimator geometries (multi-pinhole, converging, and parallel) using sheet beam X-rays. They observed a minimum detection limit of 0.8 mg/mL AuNP with the parallel collimator [37]. Multiple detectors arranged in a ring surrounding the object have also been considered in simulation studies to improve the sensitivity and imaging performance of the system [38], [39]. Optimization studies on the positioning of multiple detectors have been reported to further improve the SNR based on the anisotropic angular-energy distribution of the Compton scattering photons [40], [41], [42]. Jung et al. [48] and Kim et al. [49] have developed a dynamic dual modality in-vivo XRF imaging system to obtain both the functional and anatomical information on the same imaging bed and to study the accumulation and washout biodistribution of metal nanoparticles in mice. Recently, Moktan et al. [43] and Manohar et al. [44] demonstrated a detection limit of less than 0.3 mg/mL for AuNP using a CdTe (Amptek Inc.) detector coupled with a lead collimator and cone-beam X-rays illuminating the entire 3-cm diameter phantom. A total of 330 projections were acquired by rotating the phantom and translating the detector along the X-ray beam direction, mimicking an array of detectors. A coherent way to compare the sensitivity results from different reports would be to estimate the absolute amount of metal irradiated during each of those works. For example, in the studies reported by Moktan et al. [43] and Manohar et al. [44], the irradiated volume of metal nanoparticles in the phantom study is at least 400 mm^3^, corresponding to 6 mm diameter and 1.5 cm height of the sample containers, which yields the effective mass of the lowest concentration of AuNP irradiated to be approximately 80 *μ*g and 120 *μ*g, respectively. These amounts of metal particles could still be considered relatively high for therapeutic applications [10]. Moreover, using a high-powered (125 kVp, 3000 W) X-ray source would inevitably lead to a high radiation dose to the object, limiting its potential use in routine *in vivo* studies.

This study investigated the preliminary performance of our full-ring benchtop XFET system coupled with a low-power X-ray source (90 kVp, 60 W). The primary goal of this work is to introduce, to the best of our knowledge, the world’s first benchtop XFET imaging system equipped with a full-ring of CdTe imaging spectrometers coupled to a 96-pinhole compound-eye camera design. In the current work, the preliminary results of the sensitivity and quantification capability of the system have been evaluated using pencil beam X-rays irradiating on a small animal-sized acrylic phantom. The following sections discuss and present details about the system design and the imaging study results.

## II. METHODS AND MATERIALS

### A. Overview of the XFET System

**Fig. 1.**
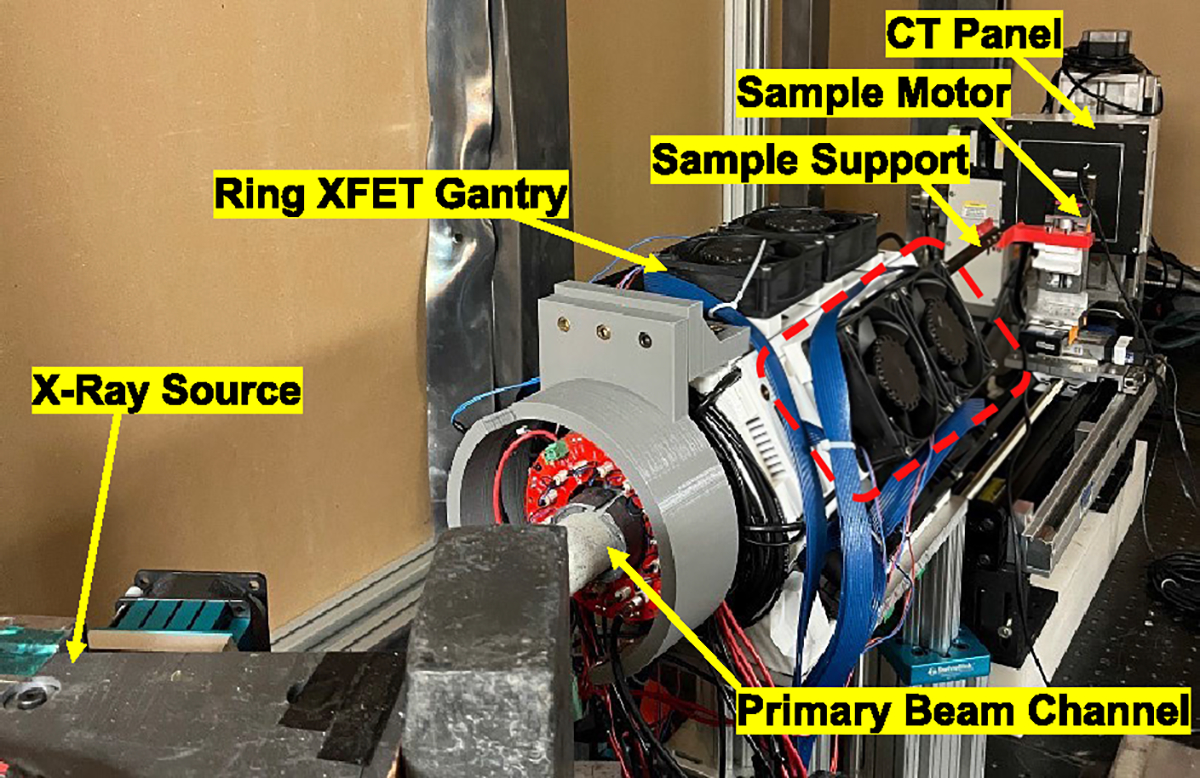
The full ring benchtop XFET system. A single detector panel is marked in the red box.

The XFET system (Fig. 1) consists of a complete ring of semiconductor detectors coupled to a polychromatic X-ray source, a computed tomography (CT) flat panel, and a sample holder and motor assembly. The detector ring is composed of 24 High Energy X-ray Imaging Technology (HEXITEC) CdTe detectors [50] arranged in a hexagonal geometry, with each side of the hexagonal ring being referred to as a detector panel, for a total detection area of 96 cm^2^. A microfocus X-ray source (DS063 Ultrabright 96000, Oxford Instruments) with a customized molybdenum target, and maximum beam rating of 90 kVp and 60 W was used to produce a polychromatic X-ray beam. The X-ray source has a beryllium window of 254 *μ*m, and its focal spot is auto-adjusted with spot size ranging from 14–20 *μ*m. Finally, an amorphous silicon-based flat panel X-ray detector (PaxScan 1313DX, Varex Imaging) with an active pixel area of 13 cm × 13 cm and 512 × 512 pixels was used to align the imaging object to the X-ray beam.

### B. The Detector Ring With 24 HEXITEC CdTe Detector Modules

**Fig. 2.**
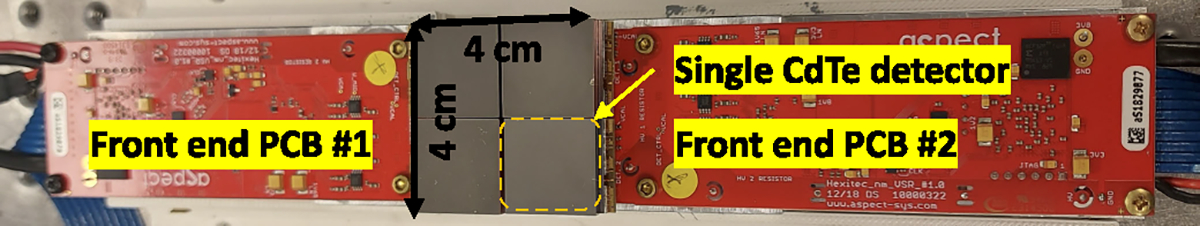
A single detector panel with four HEXITEC CdTe detector modules mounted on two analog front-end PCBs.

The detector ring comprises six detector panels. Each detector panel houses four HEXITEC CdTe detectors arranged in a 2×2 configuration (Fig. 2), with the distance between the two opposite detector panels being 82 mm. A single HEXITEC CdTe detector has an active detection area of 20 mm × 20 mm (1 mm thick), a large platinum cathode, and 80 × 80 pixelated aluminum Schottky anode with a pitch of 250 *μ*m (metal contact of 200 *μ*m × 200 *μ*m and inter-pixel gap of 50 *μ*m). The detector pixels are flip-chip bonded to the HEXITEC application-specific integrated circuit (ASIC) using gold stud and silver epoxy, and this entire assembly is referred to as a single detector module. The ASIC simultaneously reads out four 80 × 20 quadrants allowing a fast readout speed of up to 2000 frames per second. A multi-channel readout circuit optimized for the XFET application comprised 12 custom-designed printed circuit boards (PCBs), referred to as front-end PCB, connected to six remote digital data acquisition systems (DAQ) according to a modular and expandable architecture. A single front-end PCB is connected to two HEXITEC CdTe detector modules through two 34-way connectors. Two front-end PCBs are mounted on each detector panel (Fig. 2).

The front-end PCB provides HV bias and power, control logic, and digitizes the amplified analog anode signal. Two Peltier cooling units, copper heat sink, and fans (12 V, 0.5 mA) are also installed on each detector panel for cooling and heat dissipation. A more detailed information about the multi-channel readout circuitry can be found in [51].

### C. The Compound-Eye Multi-Pinhole Aperture

**Fig. 3.**
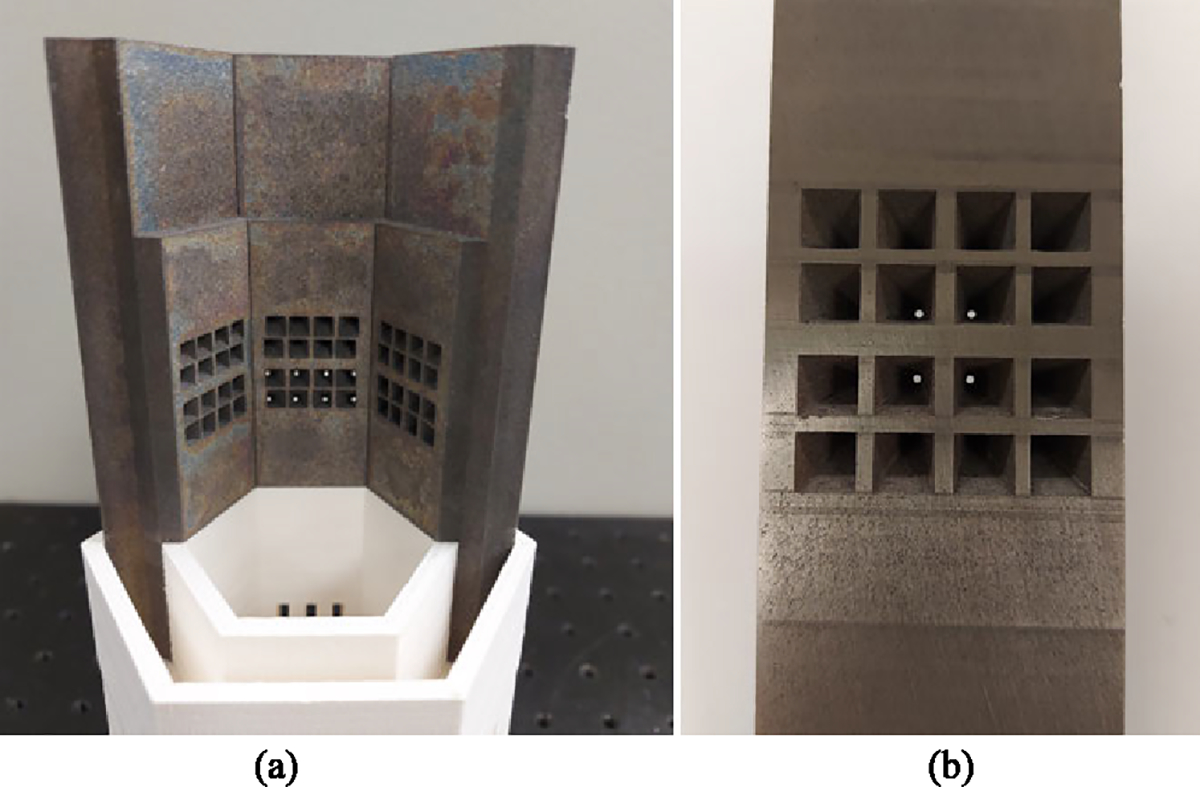
Multi-pinhole collimator. (a) Three collimator panels arranged in the hexagonal geometry (internal side). (b) Single collimator panel with 16 knife-edge pinholes of 1 mm diameter, 1:2 minification factor (external side). Each tungsten block is 12 cm long and presents a 14 mm thick central section.

In this study, the detector ring is coupled to a 96-pinhole Inverted Compound-Eye (ICE) aperture design (Fig. 3a) that we have previously proposed and evaluated for ultrahigh-sensitivity SPECT imaging [46], [47]. Each collimator panel presents 16 knife-edge pinholes of 1 mm diameter on a 12 cm long and 14 mm thick 3D printed tungsten block (Fig. 3b) for a total of 96 pinholes in the system coupled to an active detection area of 96 cm^2^. The 3D printing of the tungsten collimator was performed by M&I Materials Ltd using rapid additive manufacturing with selective laser melting of the tungsten powder. This technology is mature enough to handle 3D printing of complex geometries [52]. The design provides an ultra-high geometric sensitivity (~0.9%) while maintaining high spatial resolution in its 2 cm diameter and 1.8 cm axial field-of-view. The collimator is designed such that each pinhole projects the view of the object volume on a 1 cm × 1 cm non-overlapping subdetector active area with no multiplexing. Further information on the ICE design can be found in [46] and [47].

### D. Phantom Preparation

**Fig. 4.**
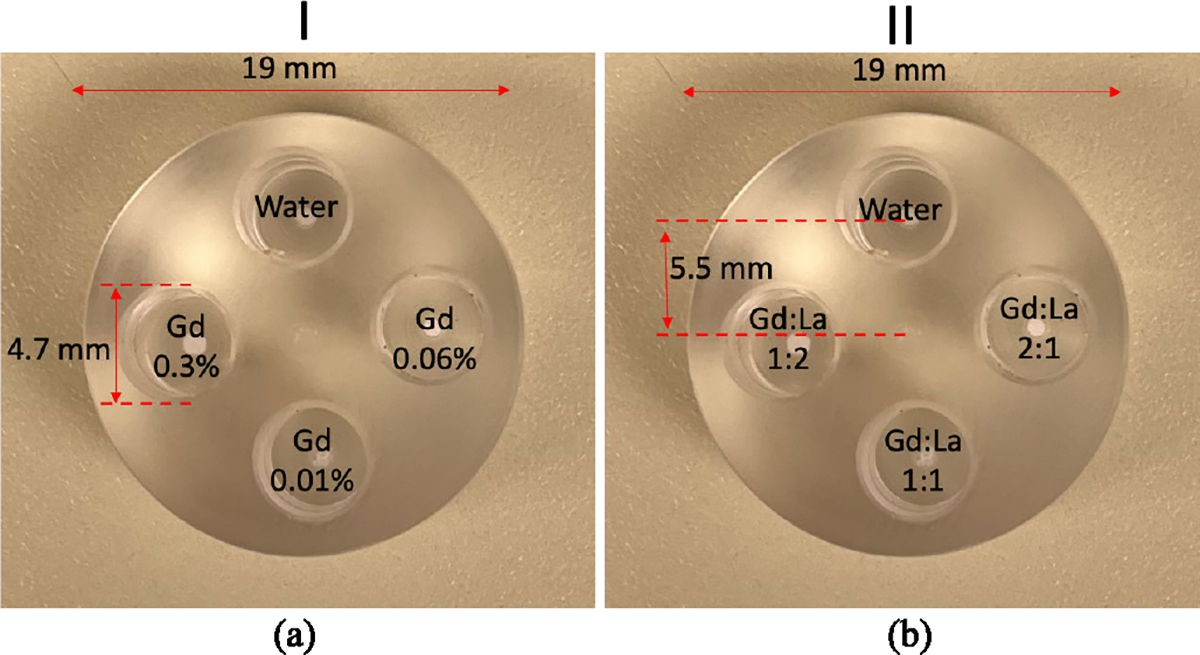
The small animal-sized cylindrical acrylic phantom of 1.9 cm diameter and 1 cm height. (a) Phantom study I: Phantom filled with three different concentrations of Gd (3 mg/mL, 0.6 mg/mL, and 0.1 mg/mL). (b) Phantom study II: Phantom study filled with mixtures of Gd and La in three different concentration ratios (1:2, 1:1, and 2:1).

In this work, a small animal-sized cylindrical acrylic phantom (density of 1.18 g/cm^3^), 19 mm in diameter and 10 mm in height, with four cylindrical holes (4.7 mm diameter and 8 mm height each) was used (Fig. 4). We performed two imaging studies with the same phantom: the first to test the detection limit of the system in the current system configuration, and the second to assess the multi-element quantification capability of the system.

In the first imaging study, referred to as *phantom study I*, three Teflon tubes (4.7 mm outer and 3 mm inner diameter) were filled with water and three different concentrations of Gd solutions (3 mg/mL, 0.6 mg/mL, and 0.1 mg/mL), as shown in Fig. 4a. In the second study, referred to as *phantom study II*, the Teflon tubes were filled with a mixture of Gd and lanthanum (La) in three different concentration ratios of 1:2, 1:1, and 2:1, respectively (Fig. 4b). The absolute values of concentrations for these mixtures were 3 mg/mL and 6 mg/mL. The center of each tube position is 5.5 mm away from the center of the phantom. The Gd and La solutions are prepared using a stock solution of Gado-DTPA (BioPAL Inc.) and LaCl_3_ powder (Sigma-Aldrich Inc.), respectively, and diluted with water to obtain the desired metal concentrations.

### E. Imaging Setup

**Fig. 5.**
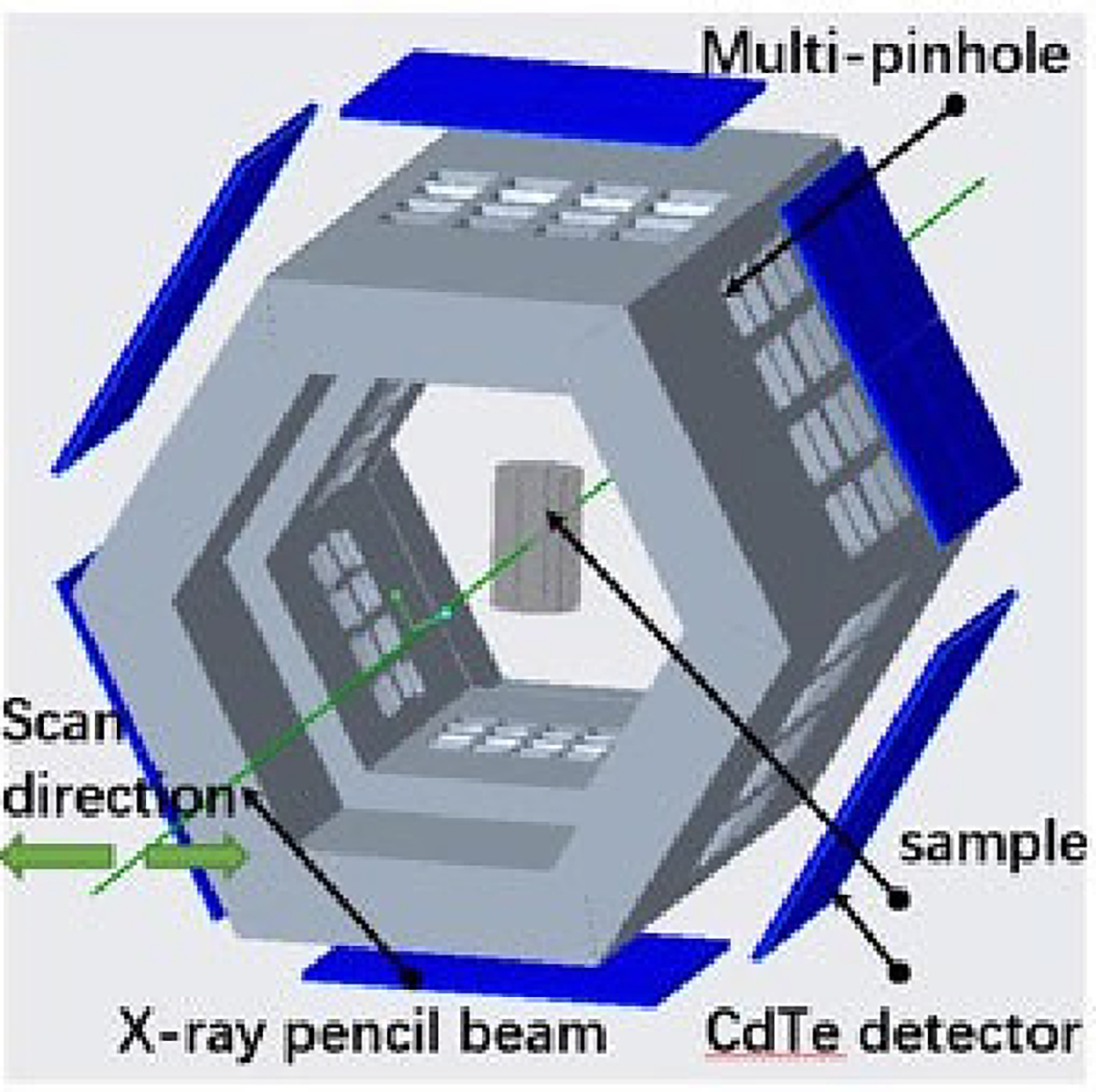
A schematic of the imaging setup with the detector and collimator panel arranged in a hexagonal ring geometry. The phantom is positioned in the center of the XFET system and is irradiated with pencil beam X-rays.

In both imaging studies, the X-ray source was operated at full power (90 kV, 60 W) and the X-ray beam was collimated using a 1-mm diameter Pt-Ir aperture inserted in a 10 mm thick lead block to produce a pencil beam of X-rays. The X-ray beam used in both studies was unfiltered. The phantom was positioned in the center of the object space of the XFET system and placed on a high-precision motorized linear stage (Newport MFA-CC) to scan through the entire phantom with a 0.5-mm step size for a total of 37 positions (Fig. 5). The acquisition time at each scan position was one hour. The linear stage was computer operated, and the data acquisition was paused during the movement of the stage.

### F. Data Processing and Image Quality

The multiplexed raw data from each CdTe detector is sent to the front-end PCB through its respective ASIC. The digitization circuit on these front-end boards digitalizes the peak-hold signal amplitude via an eight-channel analog-to-digital converter (ADC) for each detector pixel. The digitized data from the four CdTe detectors on a single detector module is transferred to a mini-PC via the remote DAQ system. The calibration coefficients and offset values for each pixel in a detector to convert the ADC units to energy are obtained by performing an energy calibration experiment using two point-sources (Am-241 with photopeak at 59.54 keV and Co-57 at 122 keV) in flat field irradiation conditions. In the current experimental study, the spectral data acquired at each scan position is processed individually. The energy-calibrated data from each pixel of each detector of the system is corrected for charge sharing by rejecting all the shared events in a 3 × 3 pixels region centered around the respective pixel [53] to obtain the charge-sharing discriminated energy (CSD) spectra.

**Fig. 6.**
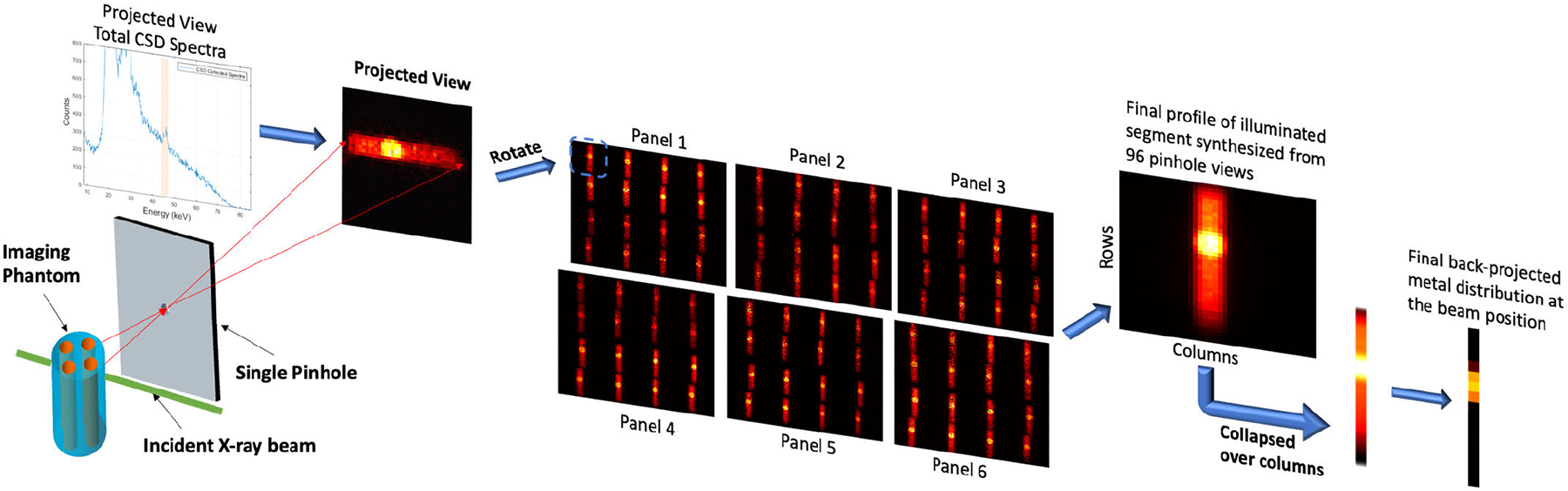
Conceptual illustration of the single view formation from the pencil beam pinhole geometry and the final elemental distribution profile synthesis from the 96 pinhole views associated with the 6 detector panels of the XFET system for a single X-ray beam position.

**Fig. 7.**
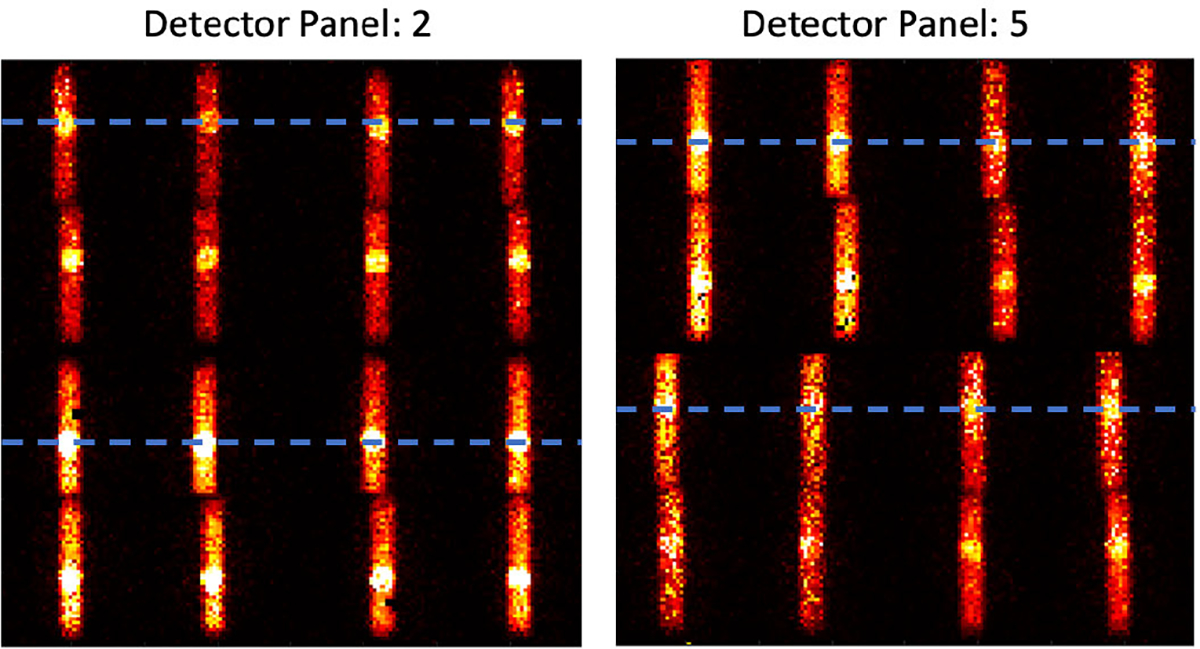
Representative raw projection from two opposite-facing detector panels of the hexagonal geometry XFET system without geometric correction of the projections. The Centre of the brightest spots are marked with a dashed line.

The image formation procedure is illustrated in Fig. 6. At each scan position, pencil beam X-rays stimulate the emission of XRF as it passes through the phantom, and 96 views (corresponding to the 96 pinholes) of the pencil-beam illuminated segment of the phantom are observed. Each pinhole on the collimator panel projects the scattered and characteristic X-ray photons from the segmented pencil beam path in the phantom onto the detector. A projected view is obtained from the CSD spectra in the energy window of interest (without Compton background subtraction) for the 1 cm × 1 cm non-overlapping subdetector area. A single projected view typically covers only a fraction of this active subdetector area. The final back-projected profile of that segment (before Compton background subtraction) was synthesized by combining the 96 pinhole views accounting for the system’s geometry. The projection of a single detector panel (4 CdTe detectors) from a representative scan position is shown in Fig. 7. As visible, the projections from two of the detectors (bottom half) are inverted because these detectors were assembled physically inverted in the system. Likewise, the projection from two opposite detector panels would have flipped views solely because they are looking at the object from opposite directions. We merge the charge-sharing corrected spectra from the 96 pinhole views by considering these geometrical arrangements and performing the necessary inversions and flips to the multiplexed data to obtain the final back-projected profile (before Compton background subtraction). This profile is then collapsed over columns without further post-processing. Note that the view at each scan position is sampled directly from the illuminated pencil beam path; thus, in principle, summing the final profile horizontally (i.e., collapsing over the columns) and extracting the net XRF signal at each row allows us to estimate the elemental distribution along the illuminated line, without having to perform a tomographic reconstruction [18], [19]. Furthermore, no attenuation correction was performed on the final back-projected image.

Due to the low penetrability of the L-shell XRF, we have formed the XFET image for both Gd and La, using the K*α* peaks (43 keV and 33 keV, respectively). A 3 keV window around the K*α* peak is defined, in the energy spectrum of each row, to extract the net XRF counts, and the background is modeled by linearly fitting the data around the close vicinity of the chosen energy window. The background-subtracted profile is then projected back onto the object space to obtain the elemental distribution in the illuminated beam path of the phantom. Finally, a 2D image of the net XRF counts was generated by scanning across the entire phantom line-by-line.

The quantitative analysis of the image is performed by evaluating the contrast-to-noise ratio (CNR). A 5 × 5 voxel region-of-interest (ROI) in the image is defined around each of the tube positions and the CNR is defined as:

(1) $$CNR=\frac{C_{tube}-C_{bkg}}{\sigma_{bkg}}$$

where, $C_{\text{tube}}$ and $C_{bkg}$ represent the average net XRF counts in a ROI defined around the tube position and around the center of the phantom, respectively. The $\sigma_{bkg}$ represents the corresponding standard deviation of counts in the ROI at the center of the phantom. Another metric utilized to assess the image quality is the signal-to-noise ratio (SNR) defined as the ratio of net XRF counts and the corresponding standard deviation of the Compton background in each image voxel. The detectability threshold as defined by the Rose criterion is selected as equal to CNR of 3 and SNR of 5 [54], [55].

### G. Cone Beam Computed Tomography

A 3D small animal micro X-ray CT (CosmoScan GX, Rigaku Corporation) with ring reduction algorithm featuring a source-to-detector distance of 203 mm and the source-to-isocenter distance of 55 mm was used to perform the CT image of the phantom. The accelerating potential of the beam was set to 90 kVp and the current to 88 *μ*A with 1-mm thick aluminum filter for a total imaging time of 840 s. The image acquisition and reconstruction were performed using the manufacture-provided software.

### H. Imaging Dose

**Fig. 8.**
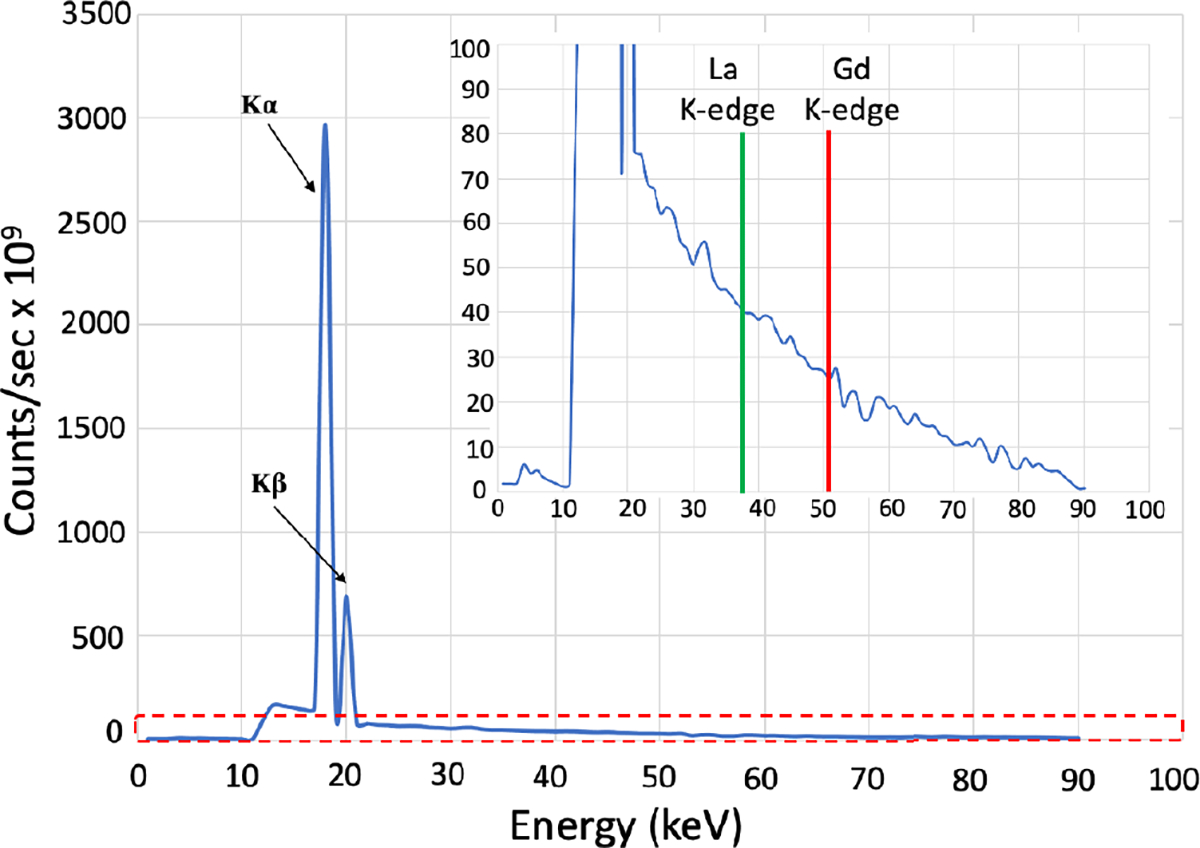
Simulated incident X-ray beam spectrum at 90 kVp and 60 W. The inset shows the zoomed in view of the region marked with red dashed box along with the k-edge energy marks of Gd and La.

A Monte Carlo (MC) model of the current XFET geometry is generated using the Geant4 toolkit [56] to assess the incident X-ray spectrum and the radiation dose at the phantom. The low energy electromagnetic physics list PENELOPE [57], [58] was used to model the bremsstrahlung and X-ray fluorescence photon production. The incident X-ray spectrum (Fig. 8) is obtained by simulating electrons (90 kVp) shooting on a molybdenum target and detecting the X-ray photons exiting a 9.5 mm diameter and 254 *μ*m thick beryllium window. From this figure, we observe that most of the incident X-ray energy is less than 21 keV which is substantially lower than the K-edge energies of Gd and La as marked on the inset. This detected X-ray spectrum is collimated to a 1-mm pencil beam and incident on the 19 mm diameter and 10 mm high small-animal sized acrylic phantom to estimate the dose delivered to the phantom during the XFET acquisition. Similarly, the dose delivered to the phantom during CT imaging was also estimated using the same Geant4 model by considering the CT acquisition geometry.

## III. RESULTS

### A. HEXITEC CdTe Detector Performance

**Fig. 9.**
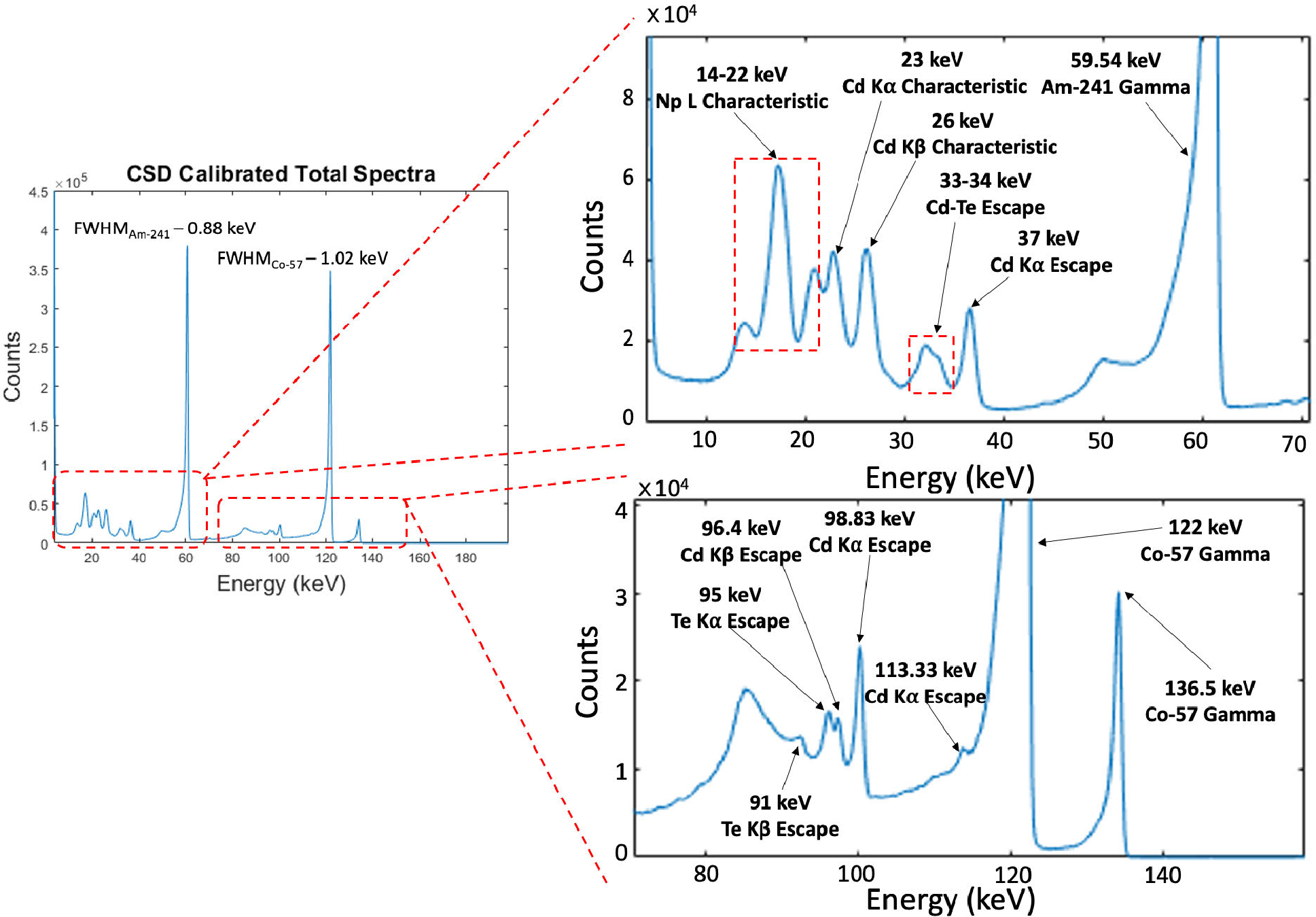
Charge sharing calibrated total spectra of the HEXITEC CdTe detector using Am-241 and Co-57 point-sources. The two subfigures are the zoomed-in view at two different regions in the spectra.

The HEXITEC CdTe detector offers an excellent energy resolution in a useful energy range of 4—150 keV for XFET applications. The spectral performance shows an energy resolution of 0.5 ± 0.03 keV FWHM (full-width-at-half-maximum) at 35 keV, 0.88 ± 0.21 keV FWHM at 60 keV and 1.02 ± 0.4 keV FWHM at 122 keV (Fig. 9). The spectrum was obtained by exposing a single HEXITEC CdTe detector to two energy calibration point-sources (Am-241 with photopeak at 59.54 keV and Co-57 at 122 keV) in flat irradiation conditions. The spectrum is corrected for charge sharing effects by rejecting all the shared events in a single frame. The zoomed views in two regions of the spectrum (Fig. 9) indicates the exceptional spectral performance of the detector, able to detect and separate primary and secondary energy peaks, including the Cd and Te escape and fluorescence peaks.

### B. Imaging Studies

**Fig. 10.**
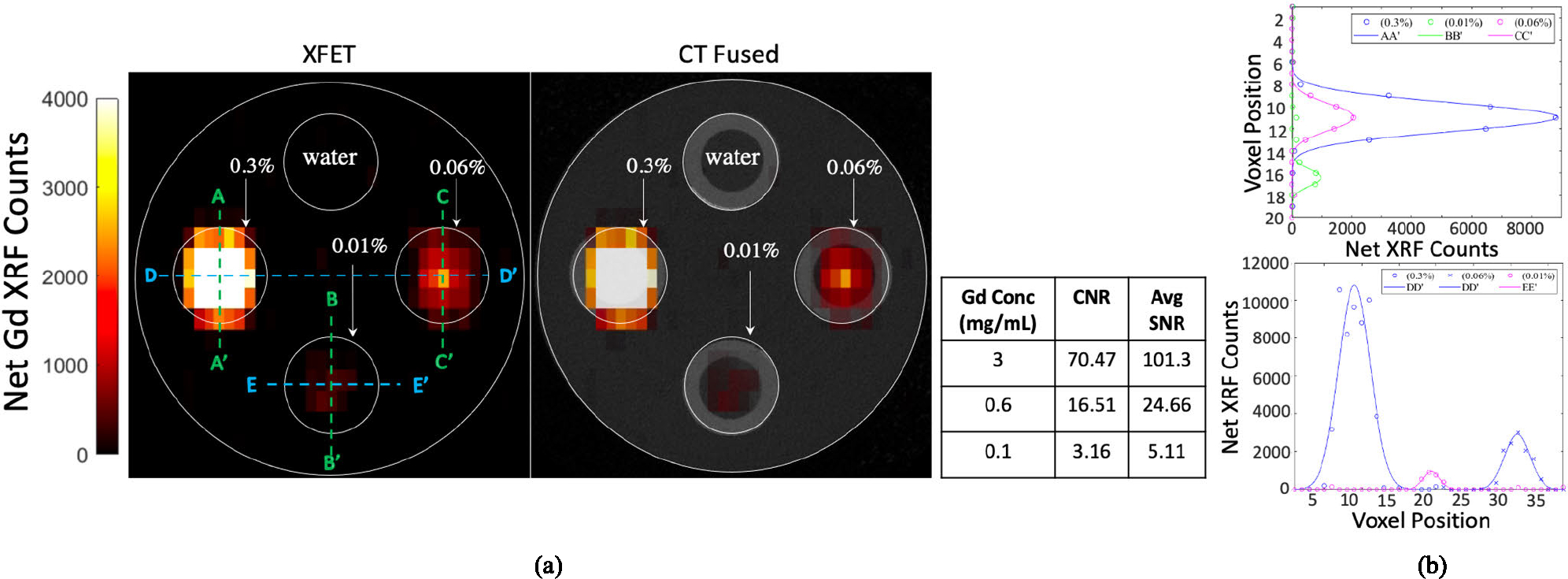
(a) XFET and the CT fused XFET Images of the (*p*hantom study I)19-mm diameter acrylic phantom with four cylindrical Teflon tubes of 4.7 mm filled with water and three different concentrations of Gd solution (3 mg/mL, 0.6 mg/mL, and 0.1 mg/mL). The table provides the CNR and the average SNR in the ROI of the three concentration tubes. (b) Cross-sectional profiles of the net XRF counts along the five different sections as labelled in the XFET image.

**Fig. 11.**
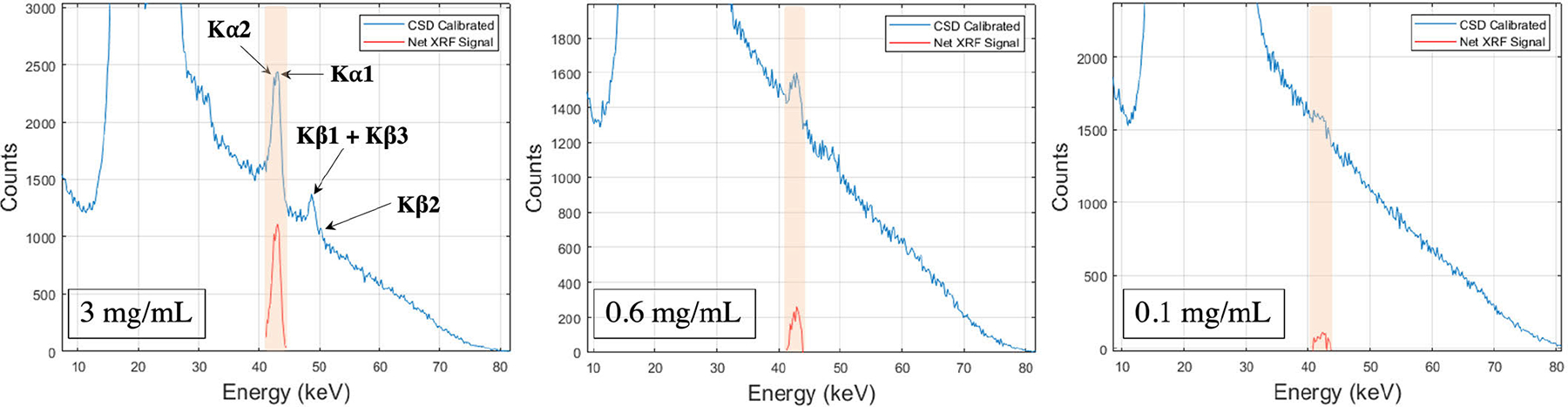
Charge sharing calibrated measured XRF spectra from the center voxel of each of the three different concentration tubes along with the background subtracted net XRF signal. The shaded region represents the energy window chosen to extract the net XRF counts.

**Fig. 12.**
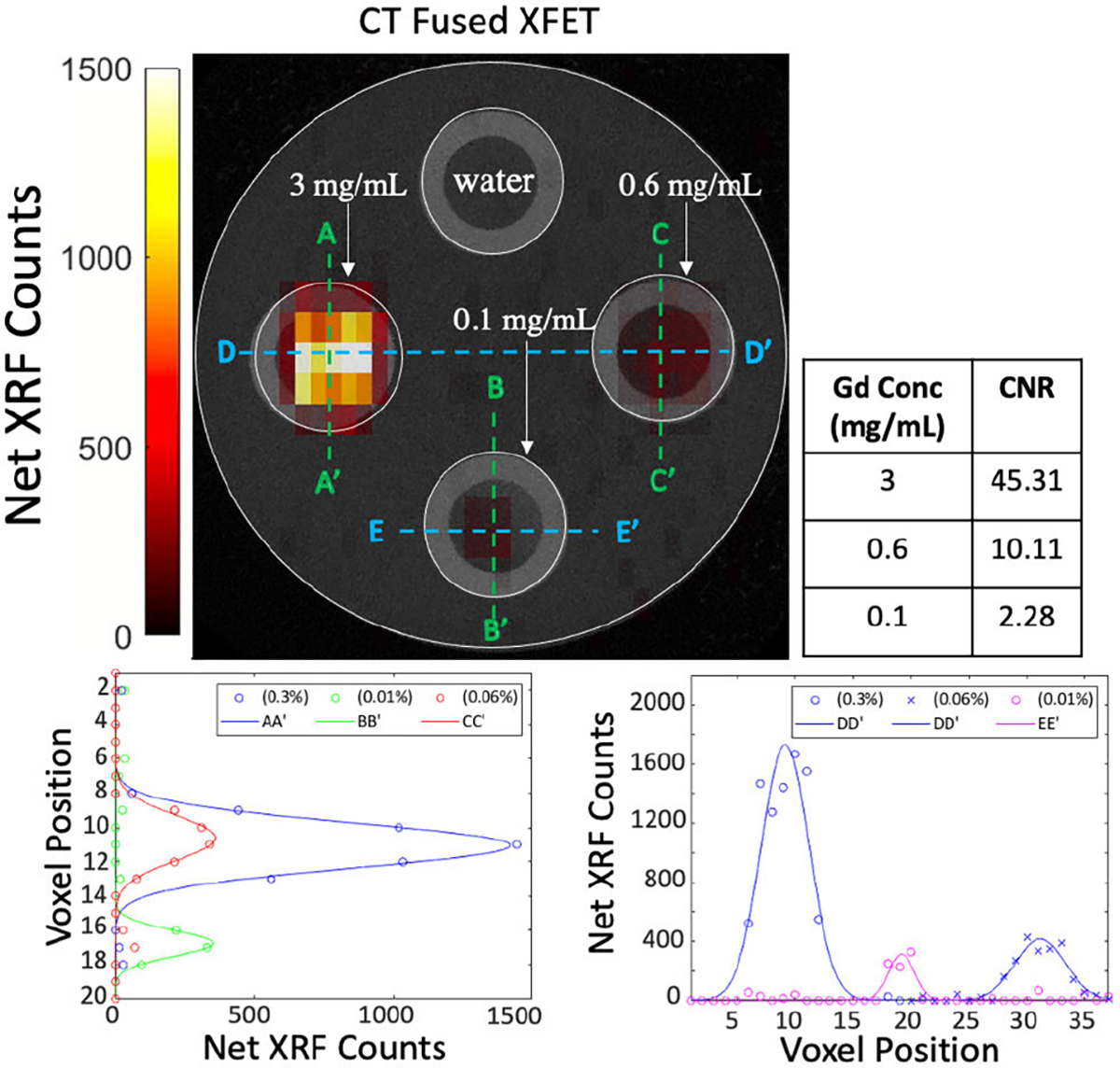
CT fused XFET Image and CNR table (*p*hantom study I)with an acquisition time of 10 minutes per scan position. Cross-sectional profiles of the net XRF counts along the five different sections as labeled in the XFET image.

The XFET image of the *phantom study I*and the CT fused image (Fig. 10a) demonstrate accurate localization of all the Gd concentration tubes in the phantom. The wall of the Teflon tubes aid in visualizing the locations of the cylindrical holes in the CT image. However, the higher density of the Teflon tube provides more attenuation and scattering compared to the acrylic phantom. The quantitative analysis of the image quality is performed by evaluating the CNR and the average SNR in the ROI of the three concentration tubes. Both the CNR and SNR values for all the Gd concentration tubes are above the detectability threshold (Fig. 10a). A more detailed analysis of the 20 × 37 pixelated (20 mm × 19 mm) XFET image is performed by observing the net XRF counts profile at five different cross-sections in the phantom (Fig. 10b). These profiles show the peak voxel position corresponding to each of the Gd concentration tubes. Qualitatively, we also observe that net XRF counts observed in the 0.1 mg/mL (0.01%) Gd concentration tube are statistically relevant. The measured XRF spectra from three different voxels each corresponding to the center position of the three Gd concentration tubes are shown in Fig. 11. In the voxel spectrum corresponding to the 3 mg/mL concentration, the different K*α* and K*β* fluorescence peaks are clearly identifiable; however, the peaks are slightly broader which could partially be attributed to the small angle scattering in the acrylic phantom. A visual inspection of the energy spectrum of the voxel corresponding to the 0.1 mg/mL Gd concentration tube indicates a well-defined XRF signal above the Compton background. In the current system configuration, thus, the lowest detected Gd concentration is 0.1 mg/mL which is equivalent to approximately 2.35 *μ*g of Gd (at the central scan position) in the irradiated volume of interest. We also test this detection limit for a shorter scan time of 10 minutes per position (Fig. 12). Although, visually differentiable the CNR for this concentration in the same 5 × 5 voxel ROI is less than 3 which could be due to a higher statistical error in the background subtracted counts around the center of the phantom. This error might arise due to larger fluctuations in the energy spectra leading to an error in Compton background estimation. Nonetheless, we do observe a reasonable number of statistically relevant counts in the cross-sectional profiles (Fig. 12) for as low as 0.1 mg/mL Gd concentration.

**Fig. 13.**
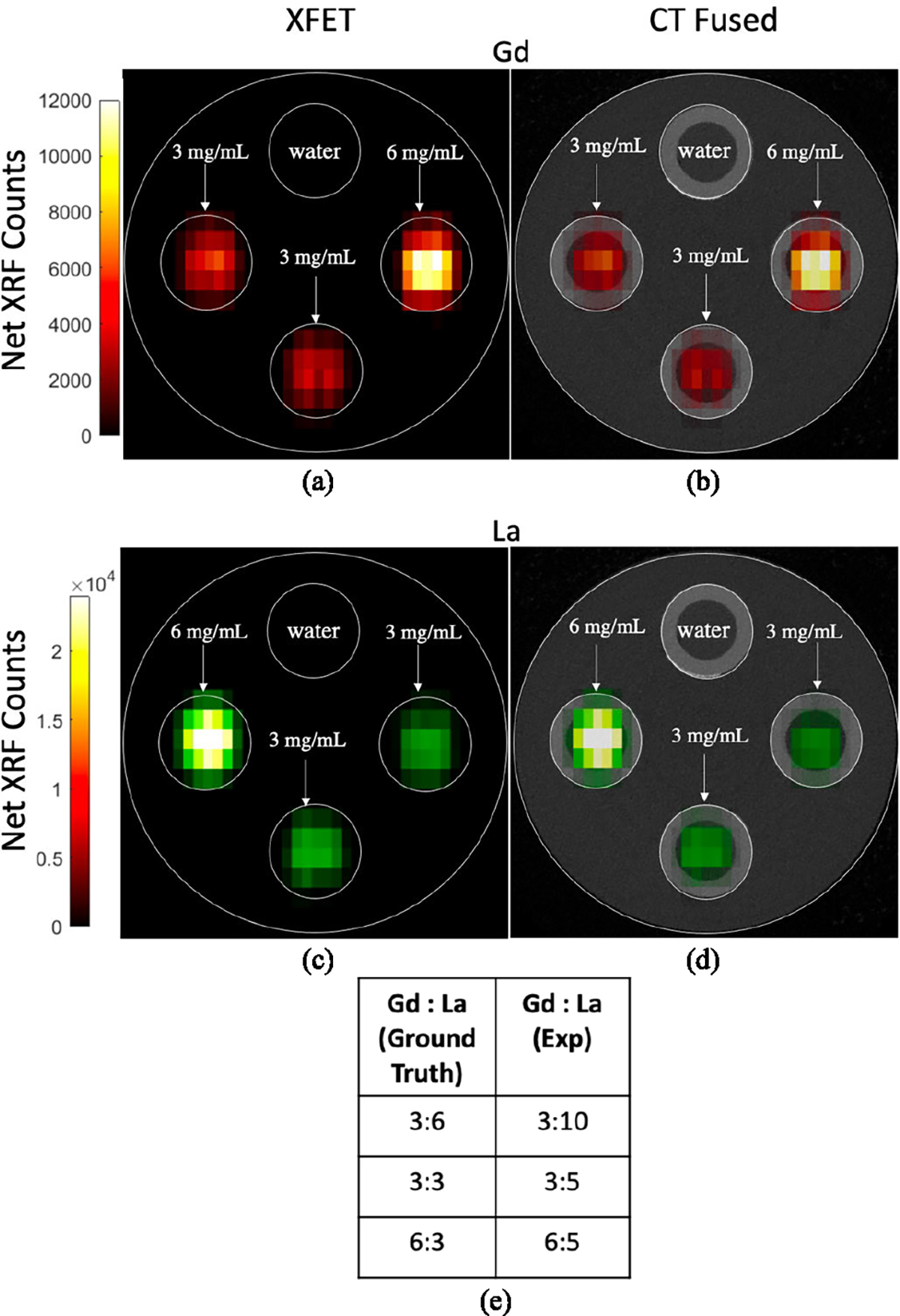
(a and b) XFET and CT fused XFET Images of the Gd, and (c and d) La for a 19-mm diameter acrylic phantom with four cylindrical Teflon tubes of 4.7 mm filled with water and Gd and La in three different concentration ratios of 3:6 mg/mL, 3:3 mg/mL and 6:3 mg/mL (*p*hantom study II). (e) The table represents the actual Gd and La concentration ratios in the phantom compared to the experimentally observed ratio quantified as the ratio of the mean XRF voxel counts of Gd and La for each tube position.

In the current effort, we also test the capability of the system to quantify the relative concentrations of different elements in a mixture. The XFET images of the *phantom study II*(Fig. 13a and c) and the corresponding CT fused images (Fig. 13 b and d) for both Gd and La again show accurate localization of the elements at the tube positions in the phantom. Qualitatively, the distribution of the Gd and La in the phantom is visually observable in the XFET images. The highest concentration spots of both Gd and La are accurately attributed to the corresponding tube positions. The quantitative measure of the concentration ratios of Gd and La is performed by taking the ratio of the mean voxel counts for both the elements at the corresponding ROI tube positions (Fig. 13e).

**Fig. 14.**
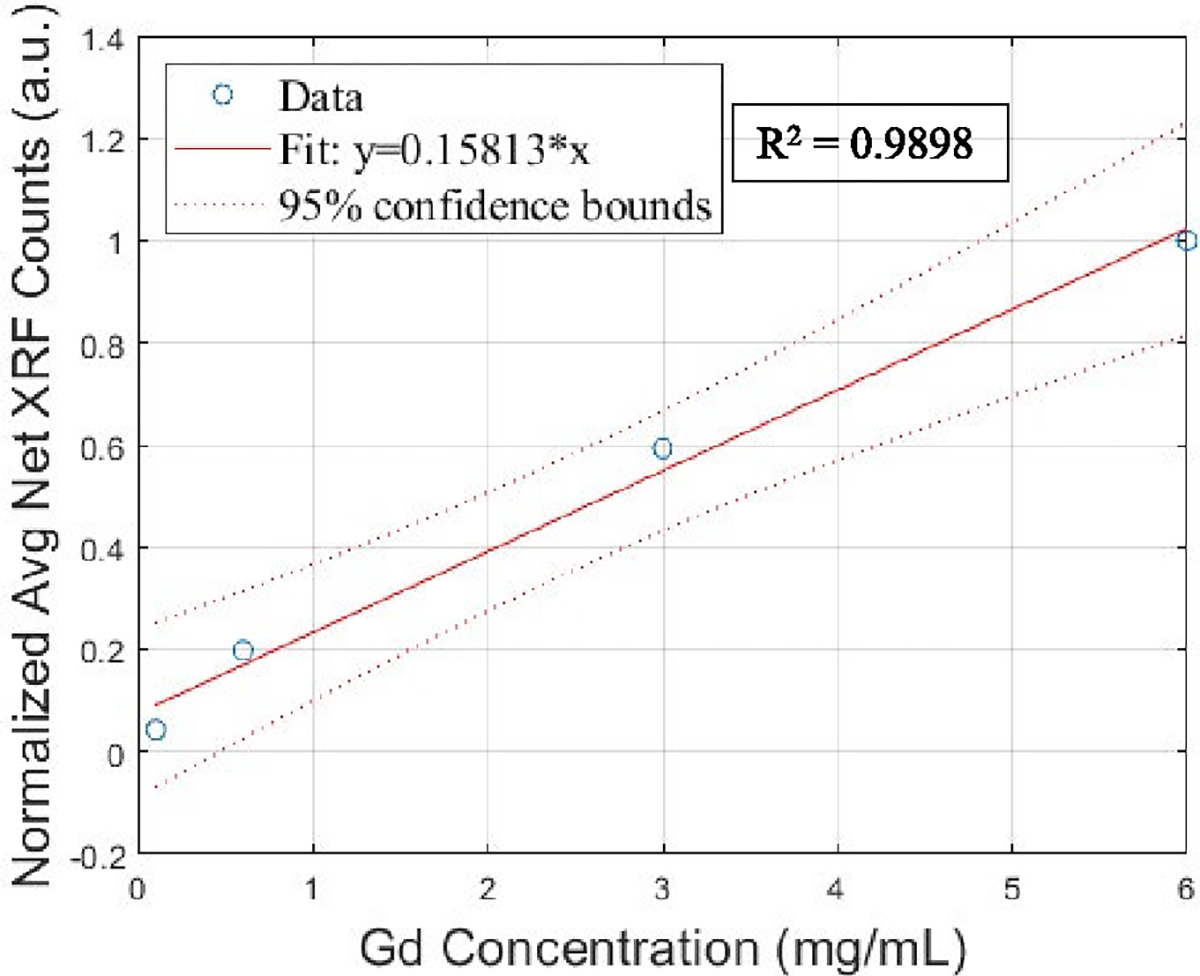
Linear relationship between the normalized average net XRF counts in the ROI for varying gadolinium concentration.

The experimentally observed concentration ratios of Gd and La compared to the actual (ground truth) concentration ratios are lower by a factor of approximately 1.67. This dissimilarity can be accounted for by considering the photoelectric cross section for La which is approximately 1.5 times higher than that for Gd at their respective K-edges [59]. The deviation can also be attributed to the difference in the intensity of the incident X-ray beam photons available at energies above the K-edge of the two elements (Fig. 8). Additionally, attenuation of the X-rays in the phantom is a major problem in the XFCT/XFET applications. Although not utilized in the current effort, an attenuation correction of the incident X-ray and the emitted XRF photons would help achieve a more accurate quantitative measure of the concentration ratios of the two elements and potentially make the image more uniform. However, we still observe a high linearity (R^2^ = 0.9898) between the normalized average net XRF counts in the ROI and the Gd concentrations for the two XFET phantom studies (Fig. 14), thereby, demonstrating the quantitative capability of the system.

We have recently developed a joint emission-attenuation estimation strategy for XFET, as detailed in [60], which in principle, should allow for improved quantitative accuracy in these XFET images.

### C. Radiation Dose

The dose rate to the acrylic phantom evaluated using the MC simulations is 16.67 cGy/min during XFET imaging at full power (90 kVp, 60 W). However, from the incident X-ray spectrum (Fig. 8), we observe that most of the incoming X-ray photons in the phantom have less than 21 keV of energy which does not contribute to the X-ray fluorescence generation and instead just induces radiation dose. Thus, we simulated the dose delivered to the phantom with a filtered X-ray spectrum (filtering all the energies lower than 21 keV) and found the dose rate to be 0.88 cGy/min. With 10 minutes of acquisition time per scan position the radiation dose is 8.8 cGy/scan position for a total of ~326 cGy of dose to the phantom during the XFET imaging. The estimated dose rate to the phantom during the CT imaging was 19.8 cGy/min. No efforts were made to optimize the CT acquisition or to reduce the dose rate in this study.

## IV. DISCUSSION AND CONCLUSION

In this paper, we have presented, to the best of our knowledge, the world’s first full-ring benchtop XFET system equipped with inverted compound-eye aperture modules coupled to a hexagonal ring of 24 HEXITEC CdTe detectors for preclinical applications. These detectors, coupled with the custom-developed multi-channel readout circuitry, offer excellent energy resolution in the useful energy range of XFET application (4—150 keV). The multi-pinhole collimator, used in the current system configuration, provides high spatial resolution with some tradeoff in system sensitivity. However, with our full-ring XFET system, we still observe a significant improvement in the detection limit of Gd (0.1 mg/mL or ~2.35 *μ*g) compared to the lowest experimental detection limit reported (80 *μ*g) [43] while not accounting for the difference in the incident X-ray power and the attenuation of the emitted XRF. We believe that the detection limit of the system presented in this study is still preliminary and that its true detection sensitivity is much higher. Theoretically, the detection limit achievable for higher Z-elements compared to Gd, such as Au or Pt, would be even better due to the higher XRF energy from these metals and the reduced attenuation in the imaging object.

We have also demonstrated the multi-element detection and quantification capability of the system. Although the observed concentration ratios of Gd and La differed from the ground truth, the difference was constant across all the tube positions. Incorporating the knowledge of the incident X-ray photon intensity above the K-edge and correcting for the difference in the photoelectric cross section of the two elements could potentially alleviate this difference, thereby facilitating more accurate quantitative imaging, desirable for the *in-vivo* applications.

The radiation dose delivered to the acrylic phantom with an acquisition time of 10 minutes per scan position and filtered incident X-ray spectrum would be ~3.26 Gy. This dose, although, is lower by a factor of two compared to the studies showing similar imaging detection limits [43], [44], is still relatively high for preclinical *in-vivo* applications [33], [48], [49]. Moreover, the long imaging time of the current system configuration further impedes its *in-vivo* applicability. The line-by-line acquisition used in this study provides a better spatial resolution with a tradeoff of radiation dose and total imaging time. Alternatively, the imaging time could significantly be improved by using a multi-slit aperture geometry. The multi-slit collimator coupled with pencil beam of X-rays offer enhanced detection limit and reduced imaging time without increasing the induced radiation dose rate [17]. Another strategy that could be employed to reduce the imaging time and radiation dose while maintaining high spatial resolution would be to perform a two-stage acquisition. The first acquisition with a slightly higher-power cone beam X-ray source to identify the ROI in the object and the second with a lower power pencil beam X-ray source imaging only around the ROI.

The XFET system presented in this paper is coupled to a low power X-ray source (90 kVp, 60 W). Alternatively, using a slightly higher powered X-ray source (as used in several other work [30], [31], [41]) would improve not only the imaging time but also the detection limit. A combination of the above-mentioned approaches would allow for a significantly improved imaging performance of the current XFET system in terms of detection limit, dose delivered as well as imaging time for future in vivo studies. The detection limit of 0.1 mg/mL Gd concentration observed in the current effort is still preliminary. Nevertheless, the detection sensitivity presented here is still biologically relevant to many *in-vivo* applications including imaging of metal-based therapeutic agents.

While we have focused on the compound-eye collimation approach in this experimental study, there are many different types of collimators involving different degrees of multiplexing in projection, such as coded apertures (i.e., uniform redundant array (URA) [61], modified uniform redundant array (MURA) [62], non-redundant array [63]) and Compton camera [64], [65], that could be explored for this application and could potentially provide improved tradeoffs between spatial resolution, sensitivity, and imaging noise. We plan to investigate these possibilities in our future studies and explore different beam configurations to optimize and demonstrate the full imaging capabilities of our benchtop XFET system.
